# Reduced vagal tone in women with the *FMR1* premutation is associated with *FMR1* mRNA but not depression or anxiety

**DOI:** 10.1186/s11689-017-9197-6

**Published:** 2017-05-02

**Authors:** Jessica Klusek, Giuseppe LaFauci, Tatyana Adayev, W. Ted Brown, Flora Tassone, Jane E. Roberts

**Affiliations:** 1 Department of Communication Sciences and Disorders, University of South Carolina, Keenan Building, Suite 300, Columbia, SC 29208 USA; 2 Department of Developmental Biochemistry, New York State Institute for Basic Research in Developmental Disabilities, 1050 Forest Hill Road, Staten Island, NY 10314 USA; 3 Department of Human Genetics, New York State Institute for Basic Research in Developmental Disabilities, 1050 Forest Hill Road, Staten Island, NY 10314 USA; 4 UC Davis MIND Institute, University of California Davis, 2825 50th Street, Sacramento, CA 95817 USA; 5 Department of Psychology, University of South Carolina, 1512 Pendleton Street, Columbia, SC 29208 USA

**Keywords:** Fragile X carriers, Vagal tone, Heart rate, Physiological arousal, FMRP, *FMR1* mRNA

## Abstract

**Background:**

Autonomic dysfunction is implicated in a range of psychological conditions, including depression and anxiety. The *fragile X mental retardation-1* (*FMR1*) premutation is a common genetic mutation that affects ~1:150 women and is associated with psychological vulnerability. This study examined cardiac indicators of autonomic function among women with the *FMR1* premutation and control women as potential biomarkers for psychological risk that may be linked to *FMR1*.

**Methods:**

Baseline inter-beat interval and respiratory sinus arrhythmia (a measure of parasympathetic vagal tone) were measured in 35 women with the *FMR1* premutation and 28 controls. The women completed anxiety and depression questionnaires. *FMR1* genetic indices (i.e., CGG repeat, quantitative FMRP, *FMR1* mRNA, activation ratio) were obtained for the premutation group.

**Results:**

Respiratory sinus arrhythmia was reduced in the *FMR1* premutation group relative to controls. While depression symptoms were associated with reduced respiratory sinus arrhythmia among control women, these variables were unrelated in the *FMR1* premutation. Elevated *FMR1* mRNA was associated with higher respiratory sinus arrhythmia.

**Conclusions:**

Women with the *FMR1* premutation demonstrated autonomic dysregulation characterized by reduced vagal tone. Unlike patterns observed in the general population and in study controls, vagal activity and depression symptoms were decoupled in women with the *FMR1* premutation, suggesting independence between autonomic regulation and psychopathological symptoms that is atypical and potentially specific to the *FMR1* premutation. The association between vagal tone and mRNA suggests that molecular variation associated with *FMR1* plays a role in autonomic regulation.

## Background

The autonomic nervous system plays a fundamental role in health. Working in conjunction with other stress regulation systems, such as the hypothalamic-pituitary-adrenal axis and the immune system, the autonomic nervous system promotes adaptability to life stressors while helping the body maintain a well-controlled, functional physiological state [1]. Optimally, the sympathetic (“fight or flight”) and parasympathetic (“rest and digest”) branches of the autonomic nervous system work together in a coordinated and often antagonistic fashion to effectively respond to internal and external demands. When the dynamic interplay between the sympathetic and parasympathetic nervous systems is functioning well, the autonomic system serves a broad protective role, boosting the immune system, shielding against cardiovascular disease, and warding away psychopathology [2]. Conversely, dysfunction of the autonomic nervous system is associated with vulnerability to a host of physical and mental health disorders.

The integrity of the autonomic system can be assessed objectively and non-invasively through peripheral measures of cardiac activity. The heart is innervated by the vagal nerve, which provides a pathway for brain-heart communication via connections in the brainstem and the sinoatrial node of the heart. The measurement of inter-beat interval (IBI; or the time between consecutive heart beats) provides an estimate of general arousal level influenced by both sympathetic and parasympathetic branches of the autonomic system [3]. A specific index of parasympathetic activity can be obtained by measuring heart rate variability patterns, which index parasympathetic influences on the heart via the vagal nerve [4]. Vagal tone can be estimated through descriptive measures of heart rate variability, as well as through the quantification of respiratory sinus arrhythmia (RSA), a measure of variability in the rise and fall of heart rate that occurs with respiration (see [5], for review).

### Cardiac autonomic dysregulation in mood and anxiety disorders

Converging empirical and theoretical evidence supports cardiac autonomic indices as objective, non-invasive markers for mood and anxiety disorders (e.g., [6–9]). Dampened vagal tone is well documented in a major depressive disorder [10–13], and vagal level has also been shown to correlate with the continuous distribution of depression symptoms in non-clinical samples (e.g., [10, 14–16]). A number of reports demonstrate that depressed individuals with low vagal tone have greater symptom severity [17] and are less likely to recover or demonstrate symptomatic improvement [18, 19], and successful treatment for depression corresponds with vagal increases [20–22], albeit with some mixed findings (e.g., [23, 24]). Reduced cardiac vagal tone is also thought to represent a physiological pathway leading to anxiety. Low vagal tone relates to anxiety symptoms in non-clinical groups [25–27], and low vagal tone has been documented extensively among individuals with anxiety disorders, including populations affected by generalized anxiety disorder, panic disorder, and post-traumatic stress disorder (see [8, 9] for review).

Psychophysiological theories of vagal tone, such as the Polyvagal Theory [28, 29] and the Neurovisceral Integration Model [30, 31], support the integral role of the parasympathetic vagal system in emotional expression and regulation, accounting for reduced vagal level in mood and anxiety disorders—clinical conditions characterized by impaired emotional regulation [32]. A large body of literature suggests a mechanistic role of vagal tone in emotional regulation, coping, and social engagement [33, 34]. According to theory, vagal control is one component of a larger central autonomic network that serves to regulate defensive social behavior. The parasympathetic vagal system, in conjunction with other mechanisms, works to inhibit sympatho-excitatory threat circuits. When the vagal system is hypoactive, the body remains in a state of hypermobilization and defense, which increases “allostatic load,” or wear and tear to the bodily system over time [35]. The ability to inhibit threat circuits via the vagus is compromised in disorders of impaired emotional regulation, such as anxiety and depression (see [36]).

### Cardiac indices as biomarkers for psychological risk

The identification of biomarkers holds promise for furthering the prevention and treatment for complex mental health conditions such as anxiety and depression. Biomarkers, or measurable, endogenous traits that mark either the risk or manifestation of psychiatric illness [37], allow clinical groups to be deconstructed at the biological level, thus yielding information relevant to (1) the development of treatments targeted towards core mechanisms rather than symptoms, (2) the stratification of biological subgroups who are mostly likely to respond to targeted interventions, and (3) the identification of individuals who are most at risk, perhaps even before the onset of clinical symptoms [38]. A number of prior studies have put forth cardiac indicators of autonomic dysfunction as potentially useful biomarkers for anxiety and depression (e.g., [39, 40]) given that they co-occur with the clinical presentation of mood and anxiety disorders, are associated with symptoms in non-clinical samples, represent heritable and stable traits, are quantitative, and can be measured non-invasively and relatively quickly [41–44]. Thus, the study of cardiac activity in relation to depression and anxiety may prove useful in understanding the biological bases of these mental health conditions.

### The *FMR1* premutation as a genetic model for psychological risk

Studying cardiac function within high-risk genetic groups can inform the intercorrelation between psychophysiological traits and unique genetic profiles. In this regard, the *fragile X mental retardation-1* (*FMR1*) premutation represents a particularly promising condition for study. This genetic condition is linked with significant psychological risk and may hold promise for uncovering genetic determinants for autonomic alterations. The *FMR1* premutation occurs when the trinucleotide (CGG) sequence on the *FMR1* gene of the X chromosome expands to 55–200 repeats [45]. This mutation is characterized by excess production of *FMR1* messenger RNA (mRNA), which causes neuronal toxicity [46–48]. The *FMR1* premutation expansion is highly prevalent, occurring in approximately 1 in 113–250 females and 1 in 250–810 males depending on ethnicity and world region [49–53]. Individuals with the *FMR1* premutation are at risk for passing the mutated gene to their children, which may undergo further expansions as it is transmitted through generations, increasing the severity of the disease. Risk for generational expansion is related to genetic factors such as CGG repeat size and the number of AGG anchors, as well as environmental factors such as maternal age [54, 55]. When the expansion extends beyond 200 CGG repeats, the gene becomes inactivated by methylation and fragile X syndrome results, a neurodevelopmental disorder affecting approximately 1 in 5000 individuals [56] that is associated with intellectual disability and autism spectrum disorder [57]. The present study focuses on women with the *FMR1* premutation, who show a well-documented psychological profile characterized by risk for depression and anxiety disorders. Research focused on women—and in particular mothers—with the *FMR1* premutation is important given that the *FMR1* premutation phenotype is associated with negative outcomes both for the affected individual as well as for their children with fragile X syndrome (e.g., [58, 59]).

Women with the *FMR1* premutation, referred to as “carriers” of fragile X, were once thought to be clinically unaffected; however, new evidence clearly supports clinical involvement in this group [60]. This includes risk for fragile X-specific conditions such as fragile X-associated primary ovarian insufficiency [61] and fragile X-associated tremor/ataxia syndrome (FXTAS), a late-onset neurodegenerative movement disorder characterized by tremors, gait ataxia, peripheral neuropathy, executive dysfunction, and cognitive decline that affects about 16% of women with the premutation [62]. A subset of women with the premutation may also present with certain cognitive deficits related to executive functioning, working memory [63], and symptoms of attention deficit-hyperactivity disorder [64], which may worsen with age [65, 66]. Social difficulties have also been documented in females with the *FMR1* premutation, such as social-language deficits [58, 67] and elevated rates of autism spectrum disorder [68]. Finally, a higher rate of immune-mediated disorders, sleep apnea, hypertension, migraines, and seizures has also been observed in individuals with the premutation (reviewed in [69]).

#### Psychological risk in the *FMR1* premutation

Elevated rates of mood and anxiety disorders are one of the earliest and most consistently documented features of the *FMR1* premutation phenotype, with the risk for these conditions increasing significantly over time during adulthood [70]. Reported lifetime rates of major depressive disorder range from 12 to 54% in females with the premutation [70–75]. Lifetime rates of any anxiety disorder ranges from 25 to 47% [72, 74, 76]. This includes elevated lifetime rates of panic disorder [74, 75], social phobia [72, 74], and post-traumatic stress disorder [74]; although, findings vary somewhat depending on sample characteristics and diagnostic instruments. Reported rates for current occurrence range from 5 to 13% for major depressive disorder [70, 75] and 13 to 50% for anxiety disorders [75–77].

Psychological risk in women with the *FMR1* premutation likely has a multifactorial basis, with both *FMR1* gene dysfunction and environmental factors, such as child-related challenges, mechanistically implicated in an additive or interactive manner. Chronic stressors associated with raising a child with a developmental disorder, such as elevated child problem behaviors, are linked to increased likelihood of anxiety disorders and major depression in women with the *FMR1* premutation [75, 78]. Yet, mental health problems in women with the *FMR1* premutation often proceed the birth of their child with fragile X syndrome [75] and women with the *FMR1* premutation who do not have a child affected by fragile X syndrome also show increased rates of psychological disorders [79], supporting genetic contributors to psychopathological risk that are independent of child-related stressors. Studies have begun to characterize specific *FMR1* genetic markers associated with psychiatric symptoms. A number of reports have documented that risk for depression in the *FMR1* premutation is highest among individuals with CGG repeat length within the midsize range [70, 75, 80, 81]. Seltzer et al. [81] also detected CGG-dependent sensitivity to the environmental context, where women with midsize CGG repeat length and above-average life stress showed greater vulnerability for depression and anxiety compared to women with higher or lower repeat lengths, whereas women with midsize CGG repeats and below-average life stress were the most resilient to depression and anxiety.

Psychological vulnerability may also be related to increased *FMR1* mRNA expression, which is present at up to eightfold normal levels and increases linearly with CGG repeat size in the premutation [82, 83]. Elevated mRNA levels were found to be associated with increased psychological symptoms and reduced amygdala activation in males with the premutation, and these associations were present even among male carriers without FXTAS, suggesting that the impact of mRNA toxicity is not exclusive to FXTAS [84, 85]. Levels of mRNA are also linked with the age of depression onset in individuals with the *FMR1* premutation, consistent with the hypothesis that mRNA toxicity builds over time, contributing to vulnerability [86]. Females may be more protected from mRNA toxicity, due to the presence of the second X chromosome. In females, a high activation ratio, or a high proportion of cells carrying the normal allele on the active X chromosome, can dilute the effects of the premutation allele [87] and has been associated with less severe clinical effects, such as lower parenting stress [88] and more typical patterns of cortisol stress responses [89]. Higher levels of mRNA are correlated with self-reported anxiety symptoms among women with the premutation, but only when the sample was restricted to women with an activation ratio of less than 0.5 [84].

Slightly reduced levels of fragile X mental retardation protein (FMRP) have also been reported among individuals with the *FMR1* premutation, particularly among individuals with high CGG repeats [90, 91]. FMRP is an mRNA-binding protein that regulates the translation of about one-third of the proteins in the pre- and post-synaptic proteomes, supporting its critical role in synaptic plasticity and the development and maintenance of neuronal circuits [92]. Its absence is thought to underlie the neurobehavioral impairments seen in the full mutation [93]. Yet, the phenotypic impact of reduced FMRP in the *FMR1* premutation is less clear. Until recently, FMRP level has been measured indirectly (e.g., by counting the percent of FMRP-positive lymphocytes, see [94]), limiting the ability to capture subtle variation in protein expression. No relationships have been detected between the percentage of lymphocytes staining positive for FMRP and psychological symptoms in males or females with the *FMR1* premutation [84]. However, new technological advances, such as the approach used in the present study, allow for quantitative measurement of actual FMRP levels in the blood and may lead to a new wave of discoveries into the role of FMRP in the clinical profile of the *FMR1* premutation. For instance, studies using quantitative FMRP have revealed relationships with neurobehavioral profiles, such as preliminary evidence of FMRP-mediated blunted amygdala responses that are associated with deficient social information processing in men [85].

Despite the documented links between *FMR1*-related variation and depression in the *FMR1* premutation, a number of studies have failed to detect molecular genetic correlates of anxiety in this group [70, 72, 77, 84, 86, 95, 96] and extant findings suggest a complex, multifactorial, epigenetic basis to anxiety symptom expression. Anxiety in women with the premutation has been linked with environmental factors such as child problem behaviors [75], with the impact of the stress of raising a child with fragile X syndrome moderated by variation on *CRHR1*, a gene involved in cortisol regulation [97]. Epigenetic changes associated with abnormal methylation have also been implicated, with one study showing that methylation of the CpG10-12 sites located at the *FMR1* intron 1 boundary predicted social anxiety with 92% sensitivity in women with the *FMR1* premutation [98]. Finally, anxiety in women with the premutation has been linked with neuroanatomical changes, specifically, with reduced hippocampal volume associated with elevated mRNA [99].

### Autonomic function in the *FMR1* premutation

Given the elevated risk for depression and anxiety in the *FMR1* premutation and documented associations with *FMR1*-associated genetic mechanisms, the *FMR1* premutation may represent a “portal” condition that can yield important information on the molecular genetic basis for autonomic alterations relevant to both individuals with and without *FMR1* mutations. This work may also inform prevention and treatment efforts specific to the *FMR1* premutation. The lack of useful biomarkers represents a critical barrier to targeted treatment for this group, given the incomplete penetrance of associated clinical effects. Should cardiac indicators account for inter-individual variability in psychological risk within this population, they may prove useful in identifying vulnerable subgroups who may benefit from targeted prevention efforts.

Although cardiac autonomic dysregulation is a robust, well-documented feature of fragile X syndrome (see [100], for review), no studies have examined cardiac autonomic integrity in the *FMR1* premutation. Yet, the clinical effects of the *FMR1* premutation are highly suggestive of autonomic impairment, such as increased rates of thyroid disorders, fibromyalgia, and hypertension—all conditions associated with autonomic dysfunction [101]. Moreover, symptoms consistent with autonomic dysfunction are common in FXTAS such as impotence, bowel and bladder incontinence, hypertension, and syncope [102]. Neuropathological involvement in the autonomic ganglion of the heart and autonomic neurons of the spinal cord has also been detected in postmortem studies of individuals with FXTAS [103, 104]. In the only study to date that employed direct measures of autonomic function in the premutation, Hessl and colleagues [105] detected dampened sympathetic reactivity to a social greeting task among a sample of 12 men with the premutation using measures of electrodermal response. No associations were detected between sympathetic activation and psychological symptoms or *FMR1* molecular measures (CGG repeat size and mRNA); although, conclusions were preliminary given the small sample.

### The present study

Further investigation of autonomic nervous system activity among individuals with the *FMR1* premutation will help identify biophysiological pathways rooted in *FMR1* gene dysfunction, shedding light on biomarkers that may be linked with clinical impairment in this group. This work has implications for the identification of at-risk individuals based on specific biological markers and the potential to shift treatment efforts away from symptom-based approaches to target specific underlying mechanisms. In sum, investigations into cardiac indicators of autonomic function may provide insight into the intermediate functions of the *FMR1* gene that are coupled with psychological risk. The present study addressed the following questions:

1. Do cardiac markers of autonomic function (i.e., IBI and RSA) differ between women with the *FMR1* premutation and control women at baseline? *It was hypothesized that women with the FMR1 premutation would have elevated general arousal and reduced vagal tone when compared to controls, mirroring the physiological profile seen in the full mutation.*
2. Are cardiac markers of autonomic function related to symptoms of depression and anxiety among women with the *FMR1* premutation and control women? *It was hypothesized that low baseline vagal tone and high general arousal would relate to increased psychological symptoms in both groups.*
3. Are cardiac markers of autonomic function associated with *FMR1*-related genetic variation in women with the *FMR1* premutation? *Given the lack of prior research in this area, this aim was considered exploratory and specific hypotheses regarding gene-autonomic relationships were not made.*

## Methods

### Participants

Participants included 35 women with the *FMR1* premutation and 28 control women who were enrolled in a larger study of the social-language phenotype of women with the *FMR1* premutation. Inclusionary criteria for the broader study specified that all participants were native speakers of English, were mothers, and did not have an intellectual disability (i.e., IQ composite >80 on the Kaufman Brief Intelligence Test-II; [106]). Women who were pregnant were excluded from the study to control for pregnancy-related physiological changes (e.g., [107]). The women with the *FMR1* premutation were recruited through their children, who were participating in developmental studies of children with fragile X syndrome (PI’s: Abbeduto, Roberts). Genetic status of the women with the *FMR1* premutation was confirmed through blood tests collected through this study (*n* = 31) or via medical records. The premutation was defined as an allele ranging from 55 to 200 CGG repeats on *FMR1*. Although it was beyond the scope of the present study to conduct genetic testing on control participants, 61% of controls completed genetic testing to rule out the *FMR1* premutation through dual enrollment in a related study. Control women had no known family history of fragile X-associated conditions and were mothers of typically developing children (i.e., children who had not been diagnosed or treated for any type of developmental delay or disorder, per participant report). Additionally, control women were excluded from the study if their child scored above the cut-off for autism spectrum disorder on the Social Communication Questionnaire [108]. Recruitment of controls was focused in the local community using flyers, social media, and word of mouth.

Descriptive and demographic information is presented in Table 1. The groups did not differ significantly on age, IQ, race, or household income. A higher proportion of women with the *FMR1* premutation were using psychotropic medications compared to the control women (48 vs 15%, *p* = 0.008). While the presence FXTAS was not an exclusionary criteria, none of the women reported a clinical diagnosis of FXTAS. The groups did not differ in self-reported functional symptoms of tremor measured with the Tremor Disability Questionnaire [109], *p* = 0.508. Information on menopause status was also collected from the women with the *FMR1* premutation, as autonomic changes are observed among postmenopausal women (e.g., [110]) and the *FMR1* premutation is linked with early menopause [111]. Fifty-eight percent of the women in the *FMR1* premutation group had completed menopause, defined here as the cessation of menses for >1 year. Finally, the Parenting Stress Inventory-4 [112] was administered, given the reported relationships between parenting stress and maternal psychological health in other disability groups (e.g., [113]). Parenting stress was significantly elevated in the *FMR1* premutation group (*p* = 0.001).

**Table 1:** Group characteristics

Variable	Group		
	Women with the *FMR1* Premutation (*n* = 35)	Control Women (*n* = 28)	Test of group differences (*p* value)
Age in years			
M (SD)	44.31(8.63)	41.70 (9.34)	0.251
Range	25.53–60.94	28.72–65.23	
IQ^a^			
M (SD)	104.26 (11.90)	104.57 (11.46)	0.928
Range	81.00–130.00	83.00–135.00	
Race			0.181
Caucasian	94%	85%	
African American	3%	15%	
American Indian	3%	–	
Household Income			
<20k	9%	12%	0.164
21–40k	12%	7%	
41–80k	33%	35%	
81–120k	12%	30%	
>121k	34%	11%	
Medication use			
Atypical antipsychotics	3%	–	0.008*
Classical antipsychotics	3%	–	
Antidepressants	48%	15%	
Mood stabilizers	7%	–	
Anti-anxiety	10%	–	
Stimulants	3%	4%	
Total stress percentile^b^			0.001*
M (SD)	62.12 (22.87)	34.64 (25.63)	
Range	4.00–96.00	1.00–88.00	
Tremor Disability Score^c^			0.508
M (SD)	4.30 (15.29)	2.06 (4.15)	
Range	0–77.42	0–12.90	

^a^Measured with the Kaufmann Brief Intelligence Test-II [106]

^b^Measured with the Parenting Stress Inventory-4 [112]

^c^Potential scores range from 0 to 100, with higher scores denoting greater functional disability associated with tremor

*p < 0.05

### Procedures

Assessments took place in a university laboratory setting. Baseline cardiac activity was the first assessment activity completed after consent was obtained. To control for the potential influences of circadian rhythm, assessments were conducted in the morning (generally starting at 9:00 a.m.). Participants were asked to refrain from drinking coffee for at least 1 h prior to the assessment. Procedures were approved by the Institutional Review Board of the University of South Carolina.

### Measures

#### Cardiac autonomic activity

Cardiac activity was sampled during a 5-min baseline context where participants viewed a video of ocean waves that was designed for meditation and relaxation. Participants were instructed to “sit back and try to relax.” Data were analyzed from the final 3 min of viewing, which allowed additional time for participants to “settle into” the task. Cardiac data were collected with an Actiwave Cardio monitor (CamNtech Ltd., Cambridge, UK), which samples activity via two electrodes placed on the participant’s chest and internally records the ECG signal. Data were sampled at a rate of 1024 Hz. The IBI series was extracted from the ECG signal using QRSTool [114] with a threshold detection method. CardioEdit software (Brain-Body Center, University of Illinois at Chicago) was then used to edit artifacts and arrhythmias (<5%). Mean values for RSA and IBI were then extracted using CardioBatch software (Brain-Body Center, University of Illinois at Chicago). Briefly, CardioBatch samples sequential heart periods in 250 ms epochs and uses a 21-point moving polynomial algorithm to de-trend the data [115, 116]. The data are then bandpass filtered to extract variance associated with spontaneous breathing parameters (0.12–0.40 Hz), and RSA is estimated by transforming the variance to its natural logarithm. RSA and IBI were measured from 30 s epochs and then averaged for a total mean across the 3-min baseline period.

#### Depression symptom severity

Participants completed the Beck Depression Inventory-II [117], which is a 21-item questionnaire measuring self-reported symptoms of depression occurring over the last 2 weeks. Items are designed to reflect the defining symptoms of major depressive disorder as outlined in the Diagnostic and Statistical Manual for Mental Health Disorders [118] and are tallied to create a continuous index of depression symptom severity. The Beck depression inventory-II (BDI-II) demonstrates high test-retest reliability, internal consistency, and validity estimates (e.g., [119–121]). Nine women with the *FMR1* premutation obtained a score of 14 or higher on the BDI-II, which is considered indicative of clinical depression; no control women scored within this range.

#### Anxiety symptom severity

The Beck Anxiety Inventory [122] measured self-reported generalized anxiety symptoms occurring over the past week. This 21-item questionnaire provides a total score reflecting anxiety symptom severity, aligning with the criteria outlined in the Diagnostic and Statistical Manual for Mental Health Disorders [118]. The Beck anxiety inventory (BAI) has high internal consistency, adequate test-retest reliability, and evidence supporting convergent and discriminant validity [123, 124]. Scores above 9 are considered indicative of clinically significant anxiety; 12 women with the *FMR1* premutation and 3 control women scored above this cut-off.

#### *FMR1* molecular measures

Genomic DNA was isolated from peripheral blood lymphocytes using standard methods (Qiagen, Valencia, CA). CGG repeat length was determined using polymerase chain reaction (PCR) and Southern Blot, as previously described [125, 126]. Activation ratio, or the percent of cells carrying the normal allele on the active X chromosome, was measured using an Alpha Innotech FluorChem 8800 Image Detection System [87]. Total RNA was isolated from 3 mL of blood collected in PAXgene® tubes. To determine the relative expression levels of the *FMR1* gene, qRT-PCR amplification was carried out on total RNA using custom-designed Taqman gene expression assays, for both the validated target *FMR1* gene and the reference genes (*β-*glucoronidase) in a 7900 Sequence detector (Applied Biosystems, Foster City, CA) as detailed in [87]. A quantitative index of FMRP was obtained by using a capture Luminex-based immunoassay to determine the amount of FMRP in peripheral blood lymphocytes (expressed in pg/ug of total lysate). This assay has been shown to have high accuracy with dried blood spots, peripheral lymphocytes, brain, and other human tissues [127, 128].

### Data analysis

Analyses were conducted in SAS 9.4 [129]. The data were first examined for normality. Skewedness was detected for several variables; the Box-Cox transformation [130] was applied to find the optimal normalizing transformation for IBI (*λ* = −0.50), depression symptoms on the BDI-II (*λ* = 0), anxiety symptoms on the BAI (*λ* = −0.25), CGG repeat length (*λ* = 0), activation ratio (*λ* = 1.50), and mRNA (*λ* = −1.50); the data were transformed accordingly. The remaining variables were normally distributed and did not require transformation. Transformed values were used in all analyses. Descriptive statistics were computed and are presented in Tables 2 and 3. To explore potential confounds related to menopause status, *t* tests examined differences in the cardiac indices between the subgroups of pre- and postmenopausal women. Mean RSA and IBI did not differ by menopause status in the women with the *FMR1* premutation (*p*’s >0.172). Information on menopause status was not available for the control participants.

**Table 2:** Descriptive statistics

Variable	Group	
	*FMR1* premutation	Control
IBI (untransformed) M (SD), range	816.20 (131.11), 540.27–1135.33	791.58 (137.04), 577.93–1193.69
IBI (transformed) M (SD), range	1.93 (0.01), 1.91–1.94	1.93 (0.01), 1.91–1.94
RSA M (SD), range	4.82 (1.44), 1.78–7.64	5.56 (0.97), 3.24–7.26
BDI-II (untransformed) M (SD), range	10.97 (7.84), 0–33.00	4.07 (3.66), 0–13.00
BDI-II (transformed) M (SD), range	2.56 (0.58), 1.39–3.61	1.99 (0.44), 1.38–2.83
BAI (untransformed) M (SD), range	7.96 (6.79), 0–24.00	3.72 (5.29), 0–23.00
BAI (transformed) M (SD), range	1.73 (0.35), 1.17–2.26	1.49 (0.29), 1.17–2.25

*IBI* inter-beat interval, *RSA* respiratory sinus arrhythmia, *BDI-II* Beck Depression Inventory, *BAI* Beck Anxiety Inventory

**Table 3:** Descriptive statistics: *FMR1* molecular measures in the *FMR1* premutation group

Variable	M (SD), range
CGG repeat length (untransformed)	95.81 (17.42), 64–147
CGG repeat length (transformed)	4.54 (0.18), 4.16–4.99
Quantitative FMRP	9.16 (3.99), 2.81–18.44
Activation ratio (untransformed)	0.60 (0.18), 0.10–0.90
Activation ratio (transformed)	−0.35 (0.13), −0.65 to −1.00
Messenger RNA (untransformed)	0.77 (0.19), 0.49–1.24
Messenger RNA (transformed)	0.68 (0.01), 0.68–0.70

To test the first research question, general linear regression models tested group as a predictor of IBI and RSA. Covariates in the models included age, medication use (captured as the total number of psychotropic medications used), and parenting stress level (indexed by the total stress percentile on the Parenting Stress Inventory-4 [112]); these variables have been shown to influence cardiac functioning in prior work [27, 131–133]. Cohen’s *d* effect sizes were computed for group differences [134]. In general, effect sizes of 0.32 or less are interpreted as “small,” 0.33–0.55 “medium,” and 0.56–1.20 “large” [135]. Then, a series of general linear models tested each of the cardiac variables, group, and their interaction as predictors of depression and anxiety symptoms, after controlling for age, medication use, and parenting stress level. False discovery was controlled by adjusting at the level of the model *F* test using the Benjamini-Hochberg correction procedure [136]. Interaction contrasts were estimated to determine the effect of the cardiac predictor on psychological symptoms at each level of group. Partial eta squared (*η* ^2^_p_) effect sizes were computed. In general, values of *η* ^2^_p_ at 0.01, 0.06, and 0.14 are considered “small,” “medium,” and “large,” respectively [134].

Finally, exploratory Pearson correlations were conducted between the cardiac variables and the *FMR1* molecular variables within the *FMR1* premutation group. Significant correlations were followed with more sophisticated general linear models testing the molecular genetic variable as a predictor of the cardiac outcome, controlling for age, medication use, and parenting stress level. Because of the exploratory nature of this aim, we did not attempt to adjust for multiple comparisons in these analyses. Regression models including quadratic and cubic terms were also conducted to test for non-linear associations with CGG expansion size, considering recent reports of curvilinear associations with CGG repeat length (e.g., [75, 80]).

## Results

### Descriptive statistics

Means, standard deviations, and ranges for the cardiac indices and psychological symptoms are presented in Table 2. *t* tests indicated significant group differences for these variables, with the women with the *FMR1* premutation presenting with higher levels of both depression symptoms (*t* [58.40] = 4.22, *p* < 0.001) and anxiety symptoms (*t* [53.83] = 2.74, *p* = 0.007). Table 3 presents the descriptive statistics of the *FMR1* genetic data within the *FMR1* premutation group.

### Group comparisons on cardiac indicators

**Fig. 1:**
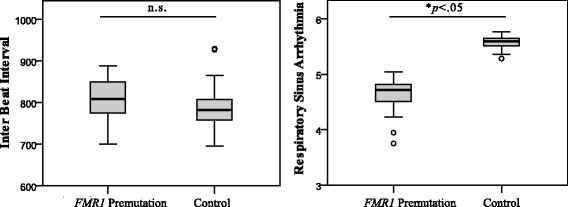
Group comparisons on respiratory sinus arrhythmia and inter-beat interval. Note: Figures present model-adjusted values, controlling for age, medication use, and parenting stress level. Untransformed IBI values are depicted for graphical representation. *Boxes* indicate data between the 25th and 75th percentile, with the *horizontal bar* reflecting the median (*whiskers* = the highest and lowest cases within the interquartile range; *open circles* = outliers, defined as cases falling greater than 1.5 times outside the interquartile range)

The combined effects of group, age, medication use, and parenting stress level accounted for significant variability in RSA, *F* (1, 51) = 2.84, *p* = 0.033, *R* ^2^ = 0.18. Group accounted for significant variability in RSA, with the women with the *FMR1* premutation exhibiting lower RSA than controls, *F* (1, 51) = 4.17, *p* = 0.046. Cohen’s *d* effect size was 0.54, consistent with a medium effect. The combined effects group, age, medication use, and parenting stress level did not account for significant variability in IBI, *F* (1, 51) = 2.54, *p* = 0.051, and *R* ^2^ = 0.17. Cohen’s *d* for the group differences in IBI was 0.05, which is consistent with a small effect size. Regression coefficients are presented in Table 4, and group comparisons are presented in Fig. 1.

**Table 4:** Regression coefficients depicting group membership as a predictor of cardiac autonomic indices

Effect	B	SE	*t*	*p*	*R* ^2^
Coefficients: RSA model					
Intercept	5.87	0.88	6.70	<0.001*	0.18
Group^a^	−0.75	0.37	−2.04	0.046*	
Age	−0.01	0.02	−0.44	0.659	
Medication use	−0.33	0.23	−1.48	0.144	
Parenting stress	<0.01	0.01	0.27	0.785	
Coefficients: IBI model					
Intercept	1.92	<0.01	466.47	<0.001*	0.17
Group^a^	<0.01	<0.01	0.16	0.876	
Age	<0.01	<0.01	2.92	0.005*	
Medication use	<0.01	<0.01	−1.11	0.272	
Parenting stress	<0.01	<0.01	0.47	0.641	

^a^The control group was set as the reference category

*p < 0.05

### Relationship between cardiac activity and symptoms of anxiety and depression

**Fig. 2:**
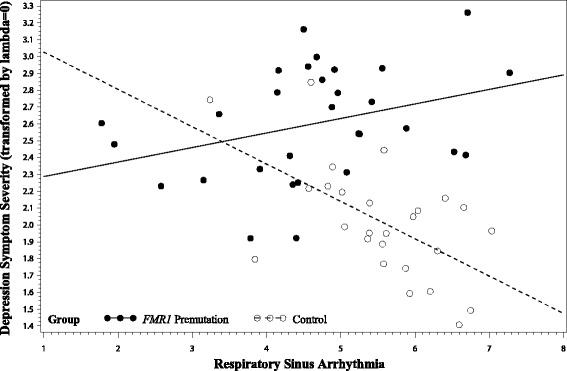
Differential associations between respiratory sinus arrhythmia and depression symptom severity across groups. Note: Model-adjusted values are depicted, controlling for age, medication use, and parenting stress level

A significant effect was detected for the overall model testing RSA as a predictor of depression symptoms (*F* [6, 48] = 9.82, *p* = 0.004, *R* ^2^ *=* 0.55). After controlling for age, medication use, and parenting stress level, the main effect for group was statistically significant, *F* [1, 48] = 21.39, *p <* 0.001, *η* ^2^_p_ = 0.31. A significant group-by-RSA interaction term was also detected (*F* [1, 48] = 7.83, *p* = 0.007, with a *η* ^2^_p_ effect size of 0.14 consistent with a large effect. Regression coefficients are presented in Table 5. Interaction contrasts confirmed that the effect of RSA on depression symptom severity differed by group; among the control women, decreased RSA was significantly associated with elevated depression symptoms with a medium-to-large effect (*F* [1, 48] = 6.40, *p* = 0.015, *η* ^2^_p_ = 0.12), whereas the association between RSA and depression symptoms was not statistically significant in the women with the *FMR1* premutation with a small effect size (*F* [1, 48] = 1.83, *p* = 0.182, *η* ^2^_p_ = 0.04), see Fig. 2. The remaining models testing the cardiac variables as predictors of depression and anxiety symptoms did not indicate a significant effect of RSA, IBI, or their interactions with group on the psychological outcomes (see Tables 5 and 6).

**Table 5:** Regression coefficients testing RSA as a predictor of depression and anxiety symptom severity

Effect	*β*	*SE*	*t*	*p*	*R* ^2^
Coefficients: Depression Symptom Severity Model					
Intercept	3.64	0.60	6.10	<0.001*	0.55
RSA	−0.24	0.09	−2.53	0.015*	
Group^a^	−1.34	0.62	−2.16	0.036*	
Group x RSA	0.32	0.11	2.85	0.007*	
Age	−0.12	0.01	−2.27	0.028*	
Medication Use	0.06	0.09	0.72	0.477	
Parenting Stress	0.01	<0.01	3.85	<0.001*	
Coefficients: Anxiety Symptom Severity Model					
Intercept	2.04	0.42	4.87	<0.001*	0.41
RSA	−0.05	0.07	−0.78	0.437	
Group^a^	−0.23	0.43	−0.52	0.604	
Group × RSA	0.07	0.08	0.88	0.384	
Age	−0.01	<0.01	−2.21	0.032*	
Medication use	0.04	0.06	0.58	0.564	
Parenting stress	<0.01	<0.01	3.06	0.004*	

^a^The control group was set as the reference category

*p < 0.05

**Table 6:** Regression coefficients testing IBI as a predictor of depression and anxiety symptom severity

Effect	*β*	*SE*	*t*	*p*	*R* ^2^
Coefficients: Depression Symptom Severity Model					
Intercept	17.76	35.52	0.50	0.619	0.48
IBI	−7.96	18.49	−0.42	0.669	
Group^a^	−31.06	43.19	−0.72	0.476	
Group × IBI	16.30	22.39	0.73	0.470	
Age	−0.02	0.01	−2.11	0.040*	
Medication use	0.04	0.09	0.40	0.691	
Parenting stress	0.01	<0.01	3.46	0.001*	
Coefficients: Anxiety Symptom Severity Model					
Intercept	15.92	22.38	0.71	0.481	0.43
IBI	−7.33	11.65	−0.63	0.532	
Group^a^	−34.14	27.09	−1.26	0.214	
Group × IBI	17.77	14.04	1.27	0.212	
Age	−0.01	0.01	−2.13	0.039*	
Medication use	0.04	0.06	0.61	0.542	
Parenting stress	0.01	<0.01	3.10	0.003*	

^a^The control group was set as the reference category

*p < 0.05

### Relationship between cardiac autonomic activity and *FMR1* molecular variation

**Fig. 3:**
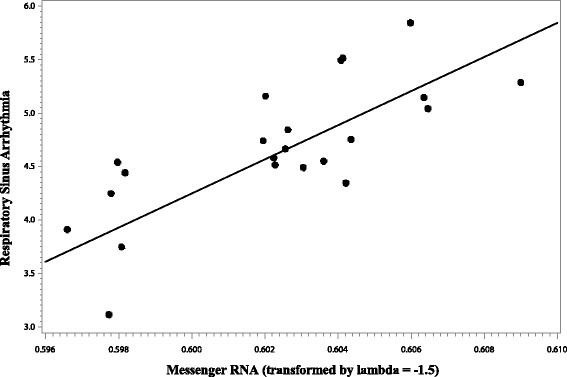
Association between *FMR1 * messenger RNA and respiratory sinus arrhythmia in women with the FMR1 premutation. Note: Model-adjusted values are presented, controlling for age, medication use, and parenting stress level

Exploratory Pearson correlations between the genetic and cardiac variables within the *FMR1* premutation group are presented in Table 7. Elevated mRNA was correlated with higher RSA (*r* = 0.51, *p* = 0.009). CGG repeat length was also positively correlated with RSA (*r =* 0.57, *p* < 0.001). Significant correlations were followed with general linear models including age, medication use, and parenting stress level as covariates. After including for these covariates, mRNA remained a significant predictor of RSA, *F* (1, 18) = 4.88, *p* = 0.040, with a *η* ^2^_p_ of 0.21 which is consistent with a large effect, see Fig. 3. The general linear model testing CGG repeat length as a predictor of RSA did not show a significant effect of CGG repeat size after controlling for age, medication use, and parenting stress level; *F* (1, 22) = 2.51, *p* = 0.128, and *η* ^2^_p_ = 0.10. Regression coefficients are presented in Table 8. Finally, general linear regression models including quadratic and cubic terms were run to test for non-linear CGG effects, with no significant non-linear CGG effects detected.

**Table 7:** Genetic correlations with the cardiac activity in women with the FMR1 premutation

	CGG repeat length	Quantitative FMRP	Messenger RNA	Activation ratio
IBI	0.14	0.26	0.17	−0.01
RSA	0.57**	−0.17	0.51**	.0.07

*IBI* inter-beat interval, *RSA* respiratory sinus arrhythmia

**p < 0.01

**Table 8:** Regression coefficients testing FMR1 mRNA and CGG repeat length as predictors of RSA

Effect	*β*	*SE*	*t*	*p*	*R* ^2^
Coefficients: *FMR1* mRNA predicting RSA					0.29
Intercept	−112.07	53.06	−2.11	0.049*	
mRNA	191.70	86.79	2.21	0.040*	
Age	0.04	0.06	0.78	0.444	
Medication use	−0.01	0.01	−0.69	0.497	
Parenting stress	−0.35	0.30	−1.17	0.256	
Coefficients: CGG repeat length predicting RSA					
Intercept	−5.74	7.76	−0.74	0.458	0.20
CGG repeat	2.52	1.59	1.58	0.128	
Age	−0.02	0.04	−0.42	0.679	
Medication use	<0.01	0.01	0.14	0.891	
Parenting stress	−0.59	0.30	−1.98	0.060	

*p < 0.05

## Discussion

Women with the *FMR1* premutation are at substantially increased risk for depression and anxiety disorders, which are conditions associated with autonomic dysregulation in the general population. Given its single-gene basis, the *FMR1* premutation may serve as a foothold to inform the genetic background for autonomic aberrations. This is the first study to examine cardiac autonomic function in women with the *FMR1* premutation and its psychological and genetic correlates. Vagal tone was significantly depressed among the women with the *FMR1* premutation, supporting impaired parasympathetic function in this group. Unlike the patterns observed in study controls and the general population, vagal tone and depression symptoms were unrelated in women with the *FMR1* premutation, suggesting that the parasympathetic system is not serving its normal emotional regulatory functions in this group. Elevated *FMR1* mRNA, which is typically associated with neuronal toxicity, was correlated with higher (i.e., “better”) vagal tone among women with the *FMR1* premutation. Results underscore the need for additional research to delineate the clinical correlates and predictive utility of autonomic markers in this high-risk group and their relationship with *FMR1-*related mechanisms.

### Group comparisons on cardiac indices of autonomic function

This study provides the first evidence of reduced vagal tone in women with the *FMR1* premutation, which could not be accounted for by elevated parenting stress or increased use of psychotropic medications. Dampened vagal tone is thought to indicate inflexibility of psychophysiological resources that regulate affective information processing [137]. A large body of literature documents a supporting role of the vagus in emotional regulation and pro-social behavior. Adults with high vagal tone show greater self-regulatory capacity [138], better regulation of negative facial expressions [139, 140], increased perceived social support [141], and increased feelings of social integration and acceptance [142]. Vagal tone has also been shown to moderate the impact of negative life experiences, acting as a buffer to shield at risk individuals from negative emotional and physical consequences [143–145]. Moreover, an “upwards spiral” reciprocal causality effect has been suggested, where high vagal tone supports psychological well-being, which in turn promotes further vagal increases [146]. The finding of dampened vagal activity among women with the *FMR1* premutation suggests that these individuals may lack the physiological resources that are needed to support optimal social-adaptive outcomes. Blunted vagal tone may be a factor in the elevated risk for emotional and physical health conditions seen in this group. Additional research is needed to determine the utility of vagal tone in predicting individual differences in clinical risk. Penetrance is not complete in the *FMR1* premutation and the identification of a biomarker that can account for phenotypic variability would contribute significantly to prevention and treatment efforts.

### Differential relationships between cardiac activity and psychological symptoms across groups

Vagal activity was not associated with depression symptoms in the women with the premutation, although, this relationship was observed in study controls and has been documented in the general population [14] and among individuals with clinically diagnosed mood disorders [10]. A similar decoupling between vagal tone and anxiety symptoms was observed in the *FMR1* premutation, which is contrary to a wealth of evidence supporting a link between vagal regulation and anxiety symptoms in other groups [8]. Together, these findings suggest that parasympathetic control of the heart via the vagal nerve is not only suboptimal (i.e., reduced in level) but also dysfunctional (i.e., not serving its normal functions) in women with the *FMR1* premutation. In other populations, the vagus is thought to play a mechanistic role in psychological vulnerability; when vagal tone is reduced, the body is unable to maintain an adaptive physiological state that promotes social engagement, leading to increased risk for emotional regulatory disorders [29]. Here, we found that vagal activity and psychological risk were not correlated in the *FMR1* premutation, despite the fact that vagal tone was reduced and psychological symptoms were increased. This may suggest different mechanistic underpinnings in the *FMR1* premutation and is consistent with distinct symptom profiles seen in this group (e.g., women with the premutation have a lower likelihood of recurrent major depressive episodes than women in the general population; Roberts et al., [75]). Future work incorporating measures of vagal reactivity may clarify relationships. The present study only included measures of tonic vagal activity, and some work suggests that task-related vagal modulation may be a more robust marker for depression than are baseline levels [39]. Future research may also investigate relationships among individuals who meet clinical thresholds for depression and anxiety, as opposed to investigating continuous symptom presentation across affected and unaffected individuals, as was done here, or among individuals with lifetime histories of depression and anxiety as opposed to current symptomatology.

### Relationship between *FMR1* molecular variation and cardiac activity

It is unexpected that elevated *FMR1* mRNA was associated with higher (i.e., “better”) vagal levels within the *FMR1* premutation group because mRNA is thought to be toxic to the neural system. So, why was elevated mRNA linked with superior vagal functioning in this sample? Undetected non-linear effects might explain this association, which would be consistent with evidence of CGG-dependent curvilinear risk patterns (i.e., [70, 75, 80, 81]) and the suggestion that maximal mRNA toxicity may occur within the mid-premutation range [80, 147]. However, an undetected curvilinear relationship seems unlikely, as the statistical tests and scatterplot distribution both suggest a linear association. The unexpected mRNA association underscores the complexity in untangling gene-brain-behavior relationships. *FMR1* mRNA toxicity is thought to involve the sequestration of other RNA binding proteins, which prevents the proteins of other genes from carrying out their normal functions [69]. Thus, *FMR1* does not function in isolation and the mechanisms by which *FMR1* variation leads to autonomic dysfunction are not straightforward. It is possible that the relationship between mRNA and vagal tone is driven by background gene dysfunction caused by protein sequestration associated with elevated mRNA. More research is needed to understand the inter-correlations between *FMR1* mRNA and other *FMR1* and non-*FMR1* mechanisms and their collective role in autonomic regulation.

Potential sex effects should also be considered, as much of our understanding of the functions of *FMR1* mRNA comes from work involving males with the premutation (e.g., [103, 148, 149]). The functions of mRNA and its neurodegenerative consequences may differ across males and females, which is consistent with evidence that FXTAS is less prevalent among females and characterized by less white matter disease, reduced brain atrophy, fewer astrocytic inclusions, and lower likelihood for dementia when compared to males [150–152]. Sex differences may be partially accounted for by random X inactivation in females, but the influence of sex-specific hormonal patterns must also be considered and has not yet been characterized. Longitudinal work will also be an informative next step, particularly given that *FMR1* mRNA gain-of-function is hypothesized to represent a degenerative, rather than developmental, mechanism, with toxicity building over time [153]. It should also be noted that mRNA levels were measured from peripheral blood lymphocytes and therefore might not necessarily reflect expression levels in relevant brain regions.

Findings did not support a relationship between cardiac activity and quantitatively measured FMRP levels in women with the premutation. No other studies have examined FMRP-autonomic relationships in the premutation, but these results are consistent with prior reports failing to detect a relationship between cardiac activity and the percent of lymphocytes staining positive for FMRP in males with the full mutation [154, 155]. One study did document a relationship between FMRP and vagal activity in females with the full mutation [155]; although, the significance of these findings are unclear as the association was only present when vagal tone was indexed using descriptive measures of heart rate variability but not when respiratory sinus arrhythmia was used, which is considered to be a more accurate measure of vagal tone [156, 157]. Overall, more research including larger samples is needed to determine whether FMRP is implicated in autonomic dysregulation in fragile X conditions.

### Summary and directions

There are a number of future directions of this work. First, follow-up studies including more diverse samples and testing gender effects are needed. This study was limited by a relatively small sample, which may have reduced statistical power. Considering that the a priori power calculations for the larger study were based on by a different set of questions and assumptions, we reported effect sizes when possible to provide insight into the strength of the detected relationships. Given the novelty of the research question addressing the relationships between cardiac function and *FMR1* molecular variation, this aim was considered exploratory and we did not attempt to correct for multiple comparisons. The exploratory associations detected here may be used to generate follow-up studies including more focused hypotheses. Follow-up work may also include more comprehensive investigation of associations with menopause, given that ~20% of women with the *FMR1* premutation experience fragile X-associated primary ovarian insufficiency, and some research suggests changes in autonomic function following menopause (e.g., [110]). Finally, genotyping was not conducted on all controls and we cannot definitively rule out the presence of atypical CGG repeat numbers in this group, which could attenuate group differences.

It should also be noted that the premutation group consisted of mothers who had a child affected by fragile X syndrome, and results may not generalize to premutation carriers who do not have an affected child. While we covaried for parenting stress levels in our models, future work may more comprehensively examine the potential moderating role of parenting stress on the patterns observed here. Interactions with environmental factors such as social support should also be considered in future work, in light of evidence suggesting an “upwards spiral” reciprocal causality effect, where vagal tone and feelings of social connectedness reciprocally and prospectively predict one another [146]. Women with the *FMR1* premutation report increased aloof personality traits [67] and heightened interpersonal sensitivity [95], which may interact with the vagal system. Vagal tone may also be important for understanding the family environment, as women with the premutation are particularly susceptible to parenting stress [158], and low vagal tone is thought to magnify sensitivity to psychosocial stressors [159]. Furthermore, vagal tone has been shown to moderate the parenting behaviors of shy-anxious mothers, influencing child outcomes [160]. Recent work shows that disruption of other allostatic systems, such as the neuroendocrine system, directly impacts maternal responsivity in mothers who carry the *FMR1* premutation [161]. Adopting a biobehavioral approach may be invaluable in parsing out the complex, multi-dimensional influences on individual and family risk factors in this population.

It should also be acknowledged that the autonomic system is one of the many bodily stress regulatory systems, and a multisystem approach is needed to account for how interactions and coordination across systems may influence findings. For instance, hypothalamic-pituitary-adrenal (HPA) axis function of the neuroendocrine system is blunted in women with the *FMR1* premutation and is related to *FMR1* variation [81, 89]. Some evidence suggests that the vagus plays an inhibitory role in the regulation of other allostatic systems, including the neuroendocrine system, with individuals with low vagal tone showing poor post-stress recovery of cardiovascular, neuroendocrine, and immune markers [162]. Better understanding of how these interacting systems function together will be important for developing targeted treatments.

## Conclusions

In summary, the present study provides evidence that autonomic dysfunction extends to the premutation, highlighting autonomic dysregulation as a hallmark feature associated with defects on *FMR1.* Associations between *FMR1*-related variation and cardiac activity were detected, which sheds light on genetic determinants of autonomic alterations relevant to *FMR1*-associated conditions and the general population as well. Despite the elevated depression and anxiety symptoms, we observed independence between psychological symptoms and the autonomic system dysfunction in women with the *FMR1* premutation group. This suggests that cardiac indices may have limited utility as biomarkers for anxiety and depression in this group. Yet, there is little understanding of the clinical consequences of autonomic dysregulation in this group and future studies may identify cardiac indices as useful markers for other clinical phenotypes associated with *FMR1* gene dysfunction, such as FXTAS*.* The identification of biomarkers for clinical risk in the *FMR1* premutation may improve early identification, tailored treatment, prevention, and the ability to predict which individuals are most at risk for late-onset symptom presentation. This study represents a first step in that direction.

## Abbreviations

BAI: Beck anxiety inventory
BDI-II: Beck depression inventory-II
*FMR1*: *Fragile X mental retardation-1*
FMRP: Fragile X mental retardation protein
FXTAS: Fragile X-associated tremor/ataxia syndrome
IBI: Inter-beat interval
mRNA: Messenger RNA
RSA: Respiratory sinus arrhythmia
